# Clinical and Molecular Diagnostic Profiling of Vaginitis Using Multiplex Real-Time PCR: A Multicenter Study

**DOI:** 10.3390/diagnostics16050783

**Published:** 2026-03-05

**Authors:** Hung Trong Mai, Chuong Canh Nguyen, Hao Thi Ngoc Vo, Thuy Thi Bich Nguyen, Trang Thi Pham, Hong Thi Ngo, Xuan Thi Ngo, Anh Thi Phuong Bui, Hue Thi Kim Ta, Anh Thi Van Nguyen

**Affiliations:** 1 Hanoi Obstetrics and Gynecology Hospital, 929 La Thanh, Lang, Hanoi 10000, Vietnam; bacsymaitronghung@gmail.com (H.T.M.); chuongnc2912@gmail.com (C.C.N.); thuya2102@gmail.com (T.T.B.N.); trangpham.hmu2589@gmail.com (T.T.P.); huettkpshn@gmail.com (H.T.K.T.); 2 ANABIO R&D Ltd. Company, No. 22, Lot 7,8 Van Khe Urban, Ha Dong, Hanoi 10000, Vietnam; vtngochao0711@gmail.com (H.T.N.V.); anhbuibio@gmail.com (A.T.P.B.); 3 Bac Ninh Center for Disease Control and Prevention, Nguyen Quyen, Vo Cuong, Bac Ninh 16000, Vietnam; ngohong13121987@gmail.com (H.T.N.); xuanngo10031976@gmail.com (X.T.N.)

**Keywords:** vaginitis, bacterial vaginosis, vulvovaginal candidiasis, sexually transmitted infections, abnormal vaginal discharge, itching, vaginal pH, multiplex real-time PCR

## Abstract

**Background:** Vaginal infections often present with overlapping symptoms and involve single or multiple pathogens. However, the relationship between clinical symptoms and molecularly defined vaginal pathogen profiles, especially in multi-pathogen infections, remains poorly characterized in a routine care setting. This study exams the connection between vaginal symptoms and pathogen profiles among women with vaginitis in Northern Vietnam. **Methods:** We conducted a multicenter cross-sectional study of women with vaginitis at Bac Ninh CDC and Hanoi Obstetrics and Gynecology Hospital between December 2023 and December 2024. Baseline demographics and clinical symptoms were assessed by physicians. Vaginal swabs were collected for pH measurement and pathogen detection using multiplex real-time PCR. The correlation was analyzed using logistic regression in GraphPad Prism v10.1.1. **Results:** Among 289 symptomatic women, abnormal vaginal discharge and itching were the most common symptoms. *Gardnerella vaginalis* was the most commonly detected pathogen, occurring alone or in combination with *Candida albicans*, *Mycoplasma hominis*, and other genital pathogens. Multi-pathogen infection was associated with abnormal vaginal discharge (OR = 5.44), itching (OR = 2.13), and elevated vaginal pH (OR = 4.70). Women at the tertiary hospital showed greater symptom burden (OR = 1.75) and higher prevalence of multi-pathogen infections (OR = 9.75) than those attending the provincial CDC. **Conclusions:** Multiplex real-time PCR combined with simple clinical indicators (symptom clustering and vaginal pH) provides practical diagnostic value for identifying multi-pathogen infections in symptomatic women. This integrated approach may support more accurate etiologic diagnosis and guide rational testing strategies, particularly in resource-limited settings.

## 1. Introduction

Vaginal infections are among the most common reasons for gynecological consultation among women of reproductive age, particularly in routine outpatient care settings, and typically present with nonspecific symptoms such as abnormal vaginal discharge or odor, itching, dysuria, or lower abdominal discomfort [1,2]. In symptomatic women, these clinical manifestations may arise from bacterial vaginosis (BV), vulvovaginal candidiasis (VVC), sexually transmitted infections (STIs), or combinations of these conditions, resulting in substantial overlap in symptom presentation [1,3]. In low- and middle-income countries (LMICs), including Vietnam, particularly in Northern Vietnam, syndromic diagnosis based primarily on symptoms alone remains predominant in routine practice, where limited accessibility to healthcare resources and non-uniform diagnostic approaches across levels of care can prevent the ability to distinguish between single-pathogen and multi-pathogen infections [4]. As a result, women with overlapping or persistent symptoms are frequently misclassified, contributing to delayed etiologic diagnosis and inappropriate treatment [5,6]. Beyond symptomatic discomfort, vaginal infections associated-BV and STIs—have been linked with adverse reproductive outcomes, including pelvic inflammatory disease, infertility, preterm birth, and increased susceptibility to HIV and other sexually transmitted infections [7]. Globally, BV is estimated to affect approximately 20–30% of women of reproductive age, while VVC occurs in the majority of women at least once during their lifetime [1,3]. In Northern Vietnam, previous studies have reported that reproductive tract infections are detected in approximately 30–50% of women presenting for gynecological care, underscoring a substantial disease burden in routine clinical practice [8,9,10].

Clinical diagnosis based solely on symptoms or routine microscopy is often unreliable in symptomatic women, owing to substantial overlap in clinical manifestations across different vaginal infections [11]. This diagnostic limitation is further compounded by the frequent occurrence of mixed vaginal infections, which are increasingly recognized as common rather than exceptional among women presenting with vaginitis symptoms. Recent advances in molecular diagnostics, notably multiplex real-time PCR assays, have enabled the simultaneous detection of multiple vaginal pathogens with high sensitivity and specificity, facilitating more comprehensive etiological assessment in symptomatic women [12,13]. In addition to improved pathogen detection, molecular assays allow semi-quantitative assessment of high-risk pathogens, which have linked to symptom severity, treatment response, and risk of recurrence. BV, VVC, and STIs are associated with a broad range of microorganisms, including bacteria, yeasts, protozoa, and viruses, such as *Gardnerella vaginalis*, *Mycoplasma hominis*, *Mycoplasma genitalium*, *Candida albicans*, *Chlamydia trachomatis*, *Neisseria gonorrhoeae*, *Trichomonas vaginalis*, and human alphaherpesvirus types 1 and 2 (HSV-1 and HSV-2) [14,15,16,17,18]. Consistent with prior reports, multiplex real-time PCR studies have highlighted the high burden of mixed infections, particularly involving *G. vaginalis*, *Candida* spp., and *Mycoplasma* spp., among symptomatic women [12,13,19]. In addition, common pathogenic features such as biofilm formation by *G. vaginalis*, vaginal dysbiosis, recurrent *Candida* infections, and increasing antimicrobial and antifungal resistance have been widely reported as contributing to disease persistence and clinical complexity in vaginitis, providing important background context for molecular diagnostic approaches [1,3,20].

While several studies have described the epidemiology of vaginal infections in selected populations, the relationships between baseline clinical symptoms and molecularly defined vaginal pathogens remain incompletely characterized in routine care. In particular, limited data are available on how infection complexity, ranging from single-pathogen to multi-pathogen detection, correlates with symptom burden and vaginal microenvironment in symptomatic women [20,21]. Previous epidemiological studies conducted in Northern Vietnam have reported a substantial burden of reproductive and lower genital tract infections among women in both community-based and clinic-based populations, especially women of reproductive age. Endogenous infections, such as BV and VVC have consistently been shown to predominate, whereas classical STIs appear to be relatively less prevalent in these clinics. These studies were conducted across both urban and sub-urban regions including Hanoi and surrounding provinces. They also highlighted heterogeneity in age distribution and healthcare-seeking behavior, as well as limitations of symptom-based diagnosis when compared with laboratory-confirmed findings [8,9,10]. In Vietnam, women with suspected vaginitis may seek care at different levels of the healthcare system depending on symptom severity, prior treatment, and referral pathways. Provincial Centers for Disease Control (CDCs) commonly manage women presenting with early or uncomplicated symptoms, whereas tertiary obstetrics and gynecology hospitals more often receive patients with persistent, recurrent, or clinically complex presentations [2,22]. These healthcare pathways provide a unique real-world context to examine the spectrum of clinical and microbiological features of suspected vaginitis across diverse care settings. Therefore, a comprehensive, multi-institutional characterization of demographics, clinical symptoms, and vaginal pathogen profiles may help clarify patterns of infection complexity and provide subsequent analyses to explore the relationship between symptoms, pathogen burden, and healthcare facilities. Accordingly, we conducted a multicenter cross-sectional study to characterize demographic features, baseline clinical symptoms, and multiplex real-time PCR-based vaginal pathogen profiles among women presenting with symptoms of vaginitis in Northern Vietnam. We further exam factors associated with infection complexity (single- vs. multi-pathogen detection) and explored whether clinical and microbiological patterns differed across healthcare settings, in order to inform context-appropriate diagnostic strategies.

## 2. Materials and Methods

### 2.1. Study Design, Participants, and Ethical Considerations

This multicenter cross-sectional study enrolled women aged >18 years old who presented with symptoms suggestive of vaginal infection at the Obstetrics and Gynecology Unit of the General Outpatient Department, Bac Ninh Center for Disease Control (Bac Ninh CDC) and the Department of Elective Gynecology, Hanoi Obstetrics and Gynecology Hospital (Hanoi OGH), over a 12-month period (25 December 2023 to 31 December 2024). Eligible participants presented with at least one clinical symptom of vaginitis, including abnormal vaginal odor, abnormal vaginal discharge, itching, burning micturition, or lower abdominal pain. Major exclusion criteria included current menstruation, pregnancy or early postpartum status, recent vaginal procedures or intravaginal medication use, and documented recent exposure to systemic or topical antibiotics, antifungals, or probiotics immediately prior to sampling. These criteria were applied to reduce major confounding effects on vaginal microbiota and molecular diagnostic results, while recognizing that earlier or undocumented antimicrobial exposure could not be fully excluded. The sample size was determined using a consecutive recruitment approach over a fixed 12-month study period, ensuring a sufficient number of observations for logistic regression analyses [23]. All eligible participants recruited at both sites underwent the same standardized clinical assessment and multiplex real-time PCR testing, without group allocation, stratification, or selective sampling.

Ethical approval was granted by the Ethics Committee of Hanoi OGH (Decision No. 2257/CNPS, 17 November 2023), and written informed consent was obtained from all participants prior to inclusion. The current analysis is limited to baseline data collected before initiation of any therapeutic intervention.

### 2.2. Clinical Data Collection and Definitions of Symptom Severity

Baseline demographic and clinical information were collected using standardized case report forms and assessed through direct physical examination by trained gynecologists. Clinical diagnosis of vaginitis subtypes was performed in accordance with international and national evidence-based guidelines (Vietnam Ministry of Health Guidelines for Reproductive Tract Infection Management, 2022; WHO Sexually Transmitted Infections Guidelines, 2021). Analyses were limited to five clinical symptoms that were assessed uniformly at both sites, including (1) abnormal vaginal odor, (2) abnormal vaginal discharge, (3) itching, (4) burning micturition, and (5) lower abdominal pain. Clinical assessments were performed in accordance with predefined diagnostic criteria [2,21] and an approved structured interview questionnaire to standardize data collection and minimize inter-observer variability. A graded symptom severity scale was not employed, as no validated scoring system is routinely implemented across the two healthcare institutions and clinician-based severity grading (e.g., mild, moderate, severe) may introduce subjectivity and assessment inconsistency. Instead, symptom severity was characterized using two complementary indicators: (1) clinical symptom burden, defined by the number of concurrent symptoms present (0–1, 2, or ≥3 symptoms), and (2) pathogen infection burden, categorized as single-pathogen versus multi-pathogen infection (≥2 detected pathogens) [24,25].

### 2.3. Vaginal Sample Collection and pH Measurement

Vaginal discharge samples were collected using polyurethane foam swabs (Henso Medical, Hangzhou, China) by gently brushing the lateral vaginal wall. The sampling procedure was performed by trained healthcare personnel following standardized protocols. Vaginal pH was measured using a calibrated handheld pH meter (Hanna Instruments, Chiba, Japan) to achieve one-decimal precision, and to improve measurement accuracy, reproducibility, and inter-site comparability compared with conventional pH indicator strips. Samples were pretreated with 1 mL of 0.9% physiological saline, followed by vigorous vortexing for 30 s. The saline solution contains no buffering, acidic, or alkaline components and therefore does not alter sample pH. An aliquot of 100 µL of the resulting suspension was applied directly to the electrode surface of the pH meter for pH determination. All measurements were performed in triplicate, and the mean pH value was used for analysis. A vaginal pH value of >4.5 was considered abnormal [2,17].

### 2.4. Multiplex Real-Time PCR Detection of Pathogen

For detection of pathogens in vaginal specimens using real-time PCR assay, total nucleic acids were extracted from 200 µL of vaginal specimens in duplicate using the QIAamp DNA Mini Kit (Qiagen, Hilden, Germany) following the manufacturer’s protocol. After extraction, 100 µL of purified DNA/RNA was divided into three aliquots (approximately 30 µL per tube) and stored at −80 °C until analysis. Vaginal pathogens were detected using an in-house multiplex real-time PCR assay previously analytically validated for sensitivity, specificity, and multiplex performance [13], including BV-associated bacteria (*Gardnerella vaginalis* (GV), *Mycoplasma hominis* (MH), and *Mycoplasma genitalium* (MG) and six pathogens associated with other STIs (Herpes simplex virus type 1 (HSV-1), HSV-2, *Candida albicans* (CA), *Trichomonas vaginalis* (TV), *Neisseria gonorrhoeae* (NG), and *Chlamydia trachomatis* (CT). The assay was organized into three multiplex panels: Panel 1 targeted GV, NG, and CT, detected using FAM, HEX, and Cy5 fluorescent dyes, respectively. Panel 2 included HSV-1 (FAM), CA (HEX), and TV (Cy5), while panel 3 targeted MH (FAM), HSV-2 (HEX), and MG (Cy5) (Supplementary Table S1). Positive control (PC) sequences for the nine STIs and the internal amplification control (IAC) were synthesized and previously cloned into the pUC19 plasmid (Supplementary Table S2) [12]. Primers and a probe specific to IAC, labeled with the ROX fluorescent dye (Invitrogen (Thermo Fisher Scientific), Singapore), were incorporated into all three panels to monitor PCR performance and rule out false-negative results. Each 25 μL real-time PCR reaction contained 5 μL template DNA, 12.5 μL 2× TOPreal qPCR PreMIX (Enzynomics Inc., Daejeon, Republic of Korea), 200 nM pathogen primers with 10–60 nM probe, 120 nM IAC primers and probe, ~1000 copies of the IAC plasmid, and nuclease-free water to volume. PCR amplification for the three panels was performed under the same following conditions: initial denaturation at 95 °C for 10 min, followed by 45 cycles of 95 °C for 15 s and 60 °C for 45 s, as previously described [13], using a 7500 real-time PCR system (Thermo Scientific, Waltham, MA, USA). A cycle threshold (C_t_) value < 40 was used to define a positive result. For each real-time PCR run, samples were processed alongside positive and negative controls, and each sample was analyzed in a single reaction.

### 2.5. Statistical Analysis

Categorical variables were summarized as frequencies and percentages and compared between groups using the Chi-square test or Fisher’s exact test, as appropriate. The Normality of continuous variables was assessed using multiple tests including the Shapiro–Wilk, D’Agostino & Pearson Anderson-Darling, and Kolmogorov–Smirnov tests. Continuous variables without normal distribution were summarized using medians and interquartile ranges (IQRs) and compared using non-parametric tests. Associations between vaginal infection complexity (single-pathogen versus multi-pathogen infection, defined as the outcome variable) and clinical characteristics, including individual symptoms (abnormal vaginal odor, abnormal discharge, itching, dysuria/frequent urination, and lower abdominal pain), symptom burden (single, two, or ≥3 concurrent symptoms), vaginal pH (3.8–4.5 versus >4.5), and healthcare setting (Bac Ninh CDC versus Hanoi OGH), were assessed through crude odds ratios calculated from 2 × 2 contingency tables and adjusted odds ratios (ORs) from logistic regression analysis with 95% confidence intervals (CIs). To account for multiple comparisons, *p*-values were adjusted using the Benjamini–Hochberg false discovery rate (FDR) procedure, where applicable. All statistical tests were two-sided, and a *p*-value < 0.05 was considered statistically significant. Statistical and graphical analyses were performed on GraphPad Prism v10.1.1 software (GraphPad Software, San Diego, CA, USA) and R statistical software (R Foundation for Statistical Computing, Vienna, Austria).

## 3. Results

### 3.1. Participant Demographics and Clinical Characteristics

During the study period, a total of 289 symptomatic women with suspected vaginitis were enrolled across the two participating healthcare facilities. As shown in Table 1, the overall mean age was 31.0 ± 9.6 years. Most participants were of reproductive age, with 91.3% aged 18–45 years and 8.7% aged 46–60 years. More than half of the cohort resided in urban areas (58.5%), while 41.5% lived in rural settings. Regarding marital status, married women accounted for the largest proportion of participants (57.4%), followed by single women (40.8%), whereas divorced women represented a small minority (1.4%). In terms of reproductive history, 44.3% of women had not given birth, 13.5% had one child, and 42.2% had two or more children. A history of HPV vaccination was reported by 17.7% of participants. With respect to genital hygiene practices, vaginal douching was reported by 14.2% of women, while the use of vaginal cleaning solutions was common, reported by 77.9% of the cohort.

Clinical symptoms were prevalent at baseline (Table 1). Abnormal vaginal discharge was the most frequently reported symptom (88.9%), followed by itching (65.1%) and abnormal vaginal odor (42.9%). Dysuria or frequent urination (13.5%) and lower abdominal pain (12.8%) were less commonly reported. Overall symptom burden was notable: 45.3% of women presented with three or more concurrent symptoms, 36.0% reported two symptoms, and 12.8% reported a single symptom. Assessment of vaginal pH revealed that the majority of participants had an elevated pH (>4.5), observed in 78.9% of cases, while only 21.1% had pH values within the normal range (3.8–4.5). Taken together, these findings indicate that the study population comprised predominantly reproductive-aged women with a high burden of vaginal symptoms and frequent vaginal pH elevation, providing a clinically relevant baseline for subsequent analyses of vaginal pathogen profiles and infection complexity.

### 3.2. Vaginal Pathogen Profile

**Figure 1:**
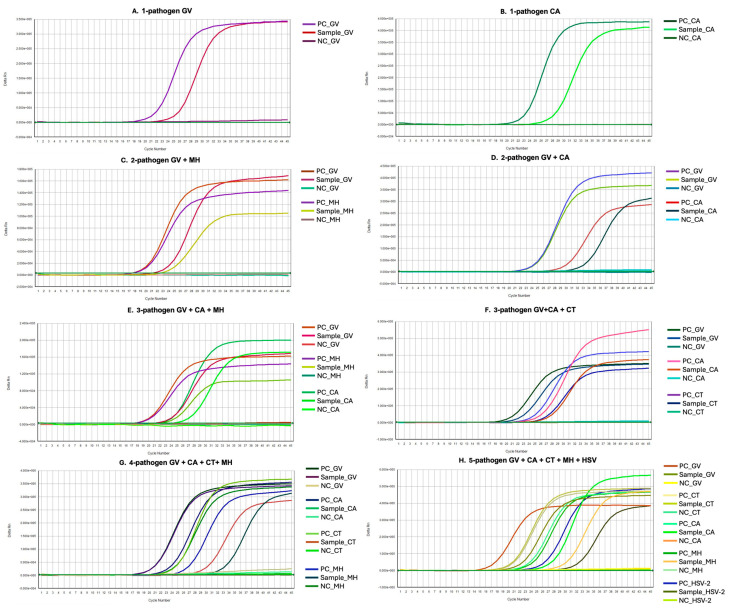
Representative multiplex real-time PCR amplification curves illustrating the detection of single- and multi-pathogen infections in vaginal swab samples from women with vaginitis. Amplification plots (Delta Rn vs. cycle number) illustrate single-pathogen detection of *G. vaginalis* and *C. albicans* (**A**,**B**), as well as examples of dual- and higher-order polymicrobial infections involving BV-associated, fungal, and STI-related pathogens within the same specimen (**C**–**H**). Overlaid amplification plots are shown for representative samples analyzed using the validated three-panel multiplex real-time PCR assay. Each curve corresponds to a fluorescence signal generated by a pathogen-specific target (FAM, HEX, or Cy5 channels). The internal amplification control (IAC; ROX) was detectable and confirmed assay performance but is not shown in the figure to maintain visual clarity in the presence of multiple target pathogen signals. Negative controls that did not generate amplification curves remained at baseline and are therefore not visually distinguishable in the plots. Abbreviations: GV: *Gardnerella vaginalis*; MH: *Mycoplasma hominis*; HSV-2: herpes simplex virus type 2; CA: *Candida albicans*; CT: *Chlamydia trachomatis*; NC: negative control; PC: positive control.

To characterize vaginal pathogen profiles among symptomatic women with suspected vaginitis, all vaginal swab samples were analyzed using a validated multiplex real-time PCR assay targeting pathogens associated with bacterial vaginosis (BV), vulvovaginal candidiasis (VVC), and sexually transmitted infections (STIs), as described in the Methods section. Overall, the majority of participants tested positive for at least one vaginal pathogen. As shown in Figure 1, the representative fluorescence amplification curves demonstrate single-pathogen detection of *Gardnerella vaginalis* or *Candida albicans* (Figure 1A,B), together with examples of dual- and higher-order polymicrobial infections involving BV-associated, fungal and STI-related pathogens co-detected within the same specimen (Figure 1C–H).

As summarized in Table 2, multiplex real-time PCR revealed a broad spectrum of vaginal pathogens and substantial heterogeneity in infection complexity within the overall cohort. Regarding BV-associated bacteria, *G. vaginalis* was by far the most frequently detected organism (89.97%), identified in the majority of symptomatic women, either as a single-pathogen infection or in combination with other vaginal or sexually transmitted pathogens. In contrast, *Mycoplasma hominis*, although the second most frequently detected BV-associated bacterium (9.69%), was rarely identified as a single-pathogen infection and occurred predominantly within multi-pathogen combinations, most often together with *G. vaginalis*. With respect to vulvovaginal candidiasis, *C. albicans* was identified in a notable proportion of women (21.8%). Importantly, *C. albicans* was commonly detected as part of mixed infections rather than as an isolated finding, and frequently co-occurring with BV-associated bacteria, particularly *G. vaginalis* and *M. hominis*. Classical sexually transmitted pathogens, including *Chlamydia trachomatis*, *Mycoplasma genitalium*, *Trichomonas vaginalis*, *Neisseria gonorrhoeae*, and herpes simplex virus types 1 and 2 (HSV-1 and HSV-2), were detected at relatively low individual frequencies within 0.69–5.54%. Nevertheless, these organisms contributed disproportionately to higher-order mixed infections, as they were rarely observed as single-pathogen infections and were almost exclusively identified in combination with other vaginal pathogens.

As for pathogen infection patterns, single-pathogen infections accounted for approximately two-thirds of cases (62.28%), predominantly driven by *G. vaginalis* detected alone. However, more than one-quarter of participants (30.45%) exhibited multi-pathogen infections, highlighting a substantial burden of concurrent microbial detection among symptomatic women. Among multi-pathogen infections, dual-pathogen infections were the most common pattern, with the combination of *G. vaginalis* and *C. albicans* accounting for the highest prevalence (15.92%), followed by progressively fewer cases involving three, four, or five concurrent pathogens. Although higher-order infections were infrequent, their presence underscores the polymicrobial nature of symptomatic vaginitis in real-world patient care and illustrates the enhanced diagnostic resolution provided by multiplex molecular testing.

Taken together, these pooled results demonstrate that suspected vaginitis among symptomatic women is characterized by both a wide etiological spectrum and marked variation in infection complexity. From isolated BV-associated bacteria to polymicrobial infections involving fungal and STI-associated pathogens, this comprehensive pathogen-level overview provides a robust microbiological foundation for subsequent analyses of clinical correlates and healthcare-setting-specific patterns.

### 3.3. Clinical Symptoms and Vaginal pH Associated with Pathogen Infection

Univariable logistic regression analysis identified several clinical features significantly associated with multi-pathogen vaginal infection (Table 3). Abnormal vaginal discharge was markedly more prevalent among women with multi-pathogen infections than those with single-pathogen infection (96.59% vs. 83.89%), corresponding to a significantly increased odds of concurrent infection (OR = 5.44; 95% CI: 1.71–17.36; *p* = 0.0022). Similarly, itching was more frequently reported in the multi-pathogen group (76.14% vs. 60%) and was positively associated with infection complexity (OR = 2.13; 95% CI: 1.21–3.72; *p* = 0.0092). After adjustment for multiple comparisons using the Benjamini–Hochberg false discovery rate procedure, the associations between multi-pathogen infection and abnormal vaginal discharge (adjusted *p* = 0.011) as well as itching (adjusted *p* = 0.023) remained statistically significant. No significant associations were observed for abnormal vaginal odor, dysuria/frequent urination or lower abdominal pain, either before or after adjustment for multiple comparisons (*p* and adjusted *p* values > 0.05). Symptom burden did not show a statistically significant association with infection complexity across categories, including women presenting with a single symptom, two concurrent symptoms, or three or more symptoms, and this remained unchanged after adjustment for multiple comparisons (*p* and adjusted *p* values > 0.05).

Vaginal pH was strongly associated with infection complexity. A normal vaginal pH range (3.8–4.5) was substantially less frequent among women with multi-pathogen infections (7.95% vs. 28.89%), indicating a protective association. In contrast, elevated vaginal pH (>4.5) was strongly associated with multi-pathogen infection (OR = 4.70; 95% CI: 2.11–11.26; *p* < 0.0001) and predominated in the multi-pathogen group.

**Figure 2:**
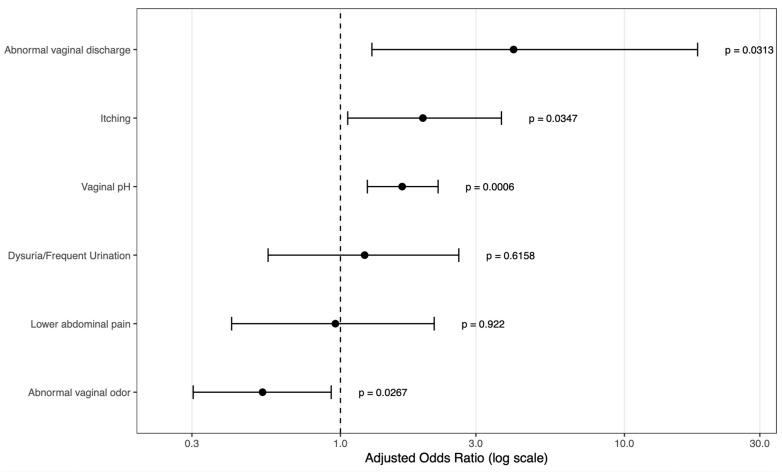
Adjusted odds ratios for factors associated with multi-pathogen infection. Forest plot showing adjusted odds ratios (aORs) and 95% confidence intervals (CIs) derived from multivariable logistic regression analysis evaluating associations between clinical symptoms and vaginal pH with the likelihood of multi-pathogen infection. Adjusted odds ratios are presented on a logarithmic scale, with the dashed vertical line indicating aOR = 1. *p* values are shown for each variable.

To evaluate the association of these clinical factors, vaginal pH, and healthcare setting to multi-pathogen infection, the final multivariable logistic regression model was fitted to only five clinical symptoms and the vaginal pH where their effects of six input variables were represented by adjusted odds ratios (see Figure 2). As for the results, abnormal vaginal discharge (aOR = 4.072; 95% CI: 1.291–18.11), itching (aOR = 1.952; 95% CI: 1.061–3.690), and elevated vaginal pH (aOR = 1.65; 95% CI: 1.244–2.208) were independently correlated with increased odds of multi-pathogen infection, whereas odor (aOR = 0.5319, 95% CI: 0.3030–0.9279) showed significant protective association. Conversely, dysuria or frequent urination, and lower abdominal pain did not exhibit independent associations with infection complexity in the adjusted model.

Collectively, these findings suggest that abnormal discharge, itching, and elevated vaginal pH may serve as practical clinical indicators for identifying women at higher probability of multi-pathogen infection, thereby supporting prioritization for multiplex molecular testing.

### 3.4. Clinical Symptoms, Vaginal pH, and Infection Profiles Associated with Study Site

To explore potential setting-related differences, we compared clinical symptoms and vaginal pathogen profiles between the two healthcare-level institutions. Comparative analyses showed significant and consistent differences in clinical manifestations, vaginal pH, and infection profiles between women recruited at Bac Ninh CDC (*N* = 128) and Hanoi OGH (*N* = 161) (Table 4). Regarding clinical symptoms, abnormal vaginal odor was significantly less prevalent among women attending Hanoi OGH compared with those at Bac Ninh CDC (39.75% vs. 71.09%), corresponding to lower odds of this symptom at the tertiary hospital (OR = 0.2683; 95% CI: 0.1626–0.4395; *p* = 0.0001). In contrast, abnormal vaginal discharge was substantially more common among women at Hanoi OGH (95.65% vs. 80.47%), with more than 5-fold higher odds compared with Bac Ninh CDC (OR = 5.34; 95% CI: 2.30–13.53; *p* = 0.0001). Following correction for multiple comparisons using the Benjamini–Hochberg false discovery rate procedure, differences between the two healthcare institutions remained statistically significant for abnormal vaginal odor and discharge (adjusted *p* = 0.00025 for both). No statistically significant differences were observed between the two sites for itching, dysuria/frequent urination, or lower abdominal pain. Analysis of symptom burden indicated that women presenting with two concurrent symptoms were more frequently observed at Hanoi OGH (41.61% vs. 28.91%), corresponding to a modest but statistically significant increase in odds (OR = 1.75; 95% CI: 1.07–2.87; *p* = 0.0254). However, this association did not remain statistically significant after adjustment for multiple comparisons using the Benjamini–Hochberg procedure (adjusted *p* = 0.0762).

In addition to symptom differences, marked differences were observed in vaginal pH distribution between the two healthcare settings. A normal vaginal pH (3.8–4.5) was substantially more common among women attending Bac Ninh CDC than among those at Hanoi OGH (39.84% vs. 6.21%). Conversely, an elevated vaginal pH (>4.5) predominated among women at Hanoi OGH (93.79%). This association was strong and statistically robust, with women at the tertiary hospital exhibiting markedly higher odds of elevated vaginal pH (OR = 10.0, 95% CI: 4.91–20.07; *p* < 0.0001).

Pronounced differences were also observed in infection profiles. *G. vaginalis* was detected in all women recruited at Bac Ninh CDC (100%), whereas a significantly lower prevalence was observed at Hanoi OGH (81.99%; *p* < 0.0001). In contrast, *M. hominis* was detected far more frequently among women attending Hanoi OGH (16.15% vs. 1.56%), corresponding to a markedly increased odds (OR = 13.10; 95% CI: 3.50–56.71; *p* < 0.0001). With respect to vulvovaginal candidiasis, *C. albicans* was detected more frequently among women attending Hanoi OGH (33.54%) compared with participants at Bac Ninh CDC (7.03%), with this difference being statistically significant (OR = 0.15; 95% CI: 0.07–0.31; *p* < 0.0001). Similarly, other sexually transmitted infections were identified only among women attending Hanoi OGH, with 18.63% of participants testing positive, whereas no cases were detected at Bac Ninh CDC. For example, *C. trachomatis* was identified exclusively at Hanoi OGH (9.94%; *p* < 0.0001), indicating a substantially higher burden of non-BV, non-VVC pathogens in the tertiary care setting. Finally, the distribution of single versus multiple infections differed strikingly between the two study sites. Nearly all women at Bac Ninh CDC presented with single-pathogen infections (91.41%), whereas multi-pathogen infections were rare (8.59%). In contrast, almost half of the women attending Hanoi OGH exhibited multi-pathogen infections (47.83%), representing a markedly higher prevalence and corresponding to substantially increased odds of multi-pathogen infection at the tertiary hospital (OR = 9.75; 95% CI: 4.92–19.31; *p* < 0.0001).

Overall, within the constraints of the present sample, women attending Hanoi OGH tended to exhibit greater clinical and microbiological complexity, reflected by a higher prevalence of elevated vaginal pH and more frequent detection of fungal, sexually transmitted, and multi-pathogen infections, whereas women at Bac Ninh CDC more commonly presented with single-pathogen infections and lower infection complexity.

## 4. Discussion

Vaginitis is a common infectious syndrome with overlapping symptoms and diverse microbial etiologies, creating persistent diagnostic challenges in routine care [1]. These diagnostic challenges have been widely recognized in the literature, as vaginitis often represents a heterogeneous syndrome involving bacterial, fungal, and sexually transmitted pathogens, with substantial symptom overlap and limited specificity of symptom-based diagnosis [1,26]. These challenges are amplified in tropical LMICs, where diagnostic practices vary widely across healthcare levels [27]. Indeed, multiple studies have demonstrated that a significant proportion of symptomatic women present with polymicrobial vaginal infections rather than isolated pathogens, reflecting complex microbial interactions within the vaginal ecosystem [28,29]. In this multi-institutional study, we show that integrating clinical indicators with multiplex real-time PCR improves detection of infection complexity beyond syndromic assessment alone. Combining vaginal pH, symptom clustering, and targeted molecular testing may therefore provide a practical and scalable diagnostic approach in routine care facilities.

The central finding of this study is the consistent association between multi-pathogen infection and increased complexity of clinical makers, particularly abnormal vaginal discharge, higher symptom burden, and elevated vaginal pH. Abnormal discharge showed the strongest association (OR = 5.44), followed by itching (OR = 2.13). Importantly, these associations remained statistically significant after adjustment for multiple comparisons, supporting the robustness of abnormal vaginal discharge and itching as independent clinical correlates of multi-pathogen infection. These results highlight the limited diagnostic specificity of individual symptoms and emphasize the value of symptom clustering rather than single-symptom interpretation, consistent with prior vaginitis research [30,31,32]. Symptom burden demonstrated a graded relationship with infection complexity. Women presenting with a single symptom were significantly less likely to harbor multi-pathogen infections, whereas two concurrent symptoms showed a borderline association. Although the association with ≥3 symptoms did not reach statistical significance in this cohort, the overall trend supports a cumulative symptom model rather than a linear symptom–pathogen relationship, which is consistent with prior observations in mixed vaginitis and polymicrobial infections [24,25,33]. Vaginal pH emerged as the strongest integrative marker of infection complexity. As a simple, low-cost bedside measurement, vaginal pH may serve as a practical triage tool in tropical and resource-limited clinics to identify women at higher risk of polymicrobial infection. A normal vaginal pH (3.8–4.5) was uncommon among women with multi-pathogen infections, whereas elevated vaginal pH (>4.5) predominated in this group, suggesting a strong association of high pH with multi-pathogen infection (OR = 4.70). This finding aligns closely with previous studies demonstrating that an elevated vaginal pH reflects disruption of *Lactobacillus*-dominated microbiota, anaerobic overgrowth, and biofilm formation characteristic of polymicrobial dysbiosis [34,35,36]. From a practical perspective, vaginal pH measurement may serve as a low-cost tool to identify patients at higher risk of mixed infections in settings where molecular testing is not immediately available. Moreover, to account for the potential confounding between clinical symptoms, the results from multivariable analysis confirm that abnormal vaginal discharge and itching, together with elevated vaginal pH, remain strongly and independently associated with multi-pathogen infection, supporting their relevance as indicators of underlying vaginal ecological disruption rather than nonspecific symptom reporting.

Across the study, *G. vaginalis* was the most frequently detected organism, appearing both as a single pathogen and as the core component of multi-pathogen combinations. This observation is consistent with the current understanding of bacterial vaginosis as a polymicrobial dysbiosis rather than a mono-etiologic infection, with *G. vaginalis* acting as a key marker organism and potential biofilm scaffold [1,37]. However, molecular detection of *G. vaginalis* does not equate to a clinical diagnosis of bacterial vaginosis per se. Rather, nucleic acid amplification tests are more appropriately interpreted as indicators of vaginal dysbiosis, as *G. vaginalis* can be detected in both symptomatic and asymptomatic women and is increasingly regarded as a marker organism within a polymicrobial biofilm ecosystem rather than a sole etiologic agent of BV [38,39,40]. Accordingly, classification of cases as “single-pathogen infection” based solely on PCR results should be interpreted cautiously in the absence of standardized clinical criteria or microscopy-based confirmation. Notably, *C. albicans* and *M. hominis* were rarely detected as isolated infections and instead occurred predominantly in combination with BV-associated bacteria, particularly *G. vaginalis* [41,42,43]. Similar co-occurrence patterns have been reported in studies from Europe and Asia, where mixed BV–VVC or BV–Mycoplasma infections were associated with broader symptom profiles and higher recurrence risk [1,44,45]. The relatively low individual prevalence of classical STI pathogens in this cohort mirrors findings from other low- and middle-income countries, yet their disproportionate contribution to higher-order mixed infections reinforces the added diagnostic value of multiplex real-time PCR approaches [12,13,19]. Similarly, detection of ≥2 pathogens by multiplex real-time PCR should not be automatically interpreted as a clinically meaningful mixed infection. Molecular assays identify pathogen nucleic acids and may reflect co-colonization or an underlying dysbiotic vaginal state rather than concurrent active infections requiring combination therapy. Several vaginal microorganisms, including *G. vaginalis*, *Mycoplasma* spp., and *Candida* spp., may be detected in asymptomatic or subclinical states, underscoring the need for careful clinical correlation when interpreting multiplex PCR results [46].

The strong association between symptom burden, elevated vaginal pH, and multi-pathogen infection highlights the limitations of syndromic diagnosis alone, particularly in women with overlapping or persistent symptoms. Multiplex nucleic acid amplification tests offer a clear advantage by enabling simultaneous detection of multiple pathogens from a single specimen, thereby reducing diagnostic uncertainty and inappropriate empiric therapy [36,37,41,42]. Based on our findings, a severity-guided diagnostic approach may be most appropriate. Women presenting with limited symptoms and a normal vaginal pH may be adequately managed with targeted testing for predominant pathogens (e.g., *G. vaginalis* and *C. albicans*), whereas those with multiple symptoms and elevated vaginal pH are more likely to benefit from comprehensive multiplex testing for common pathogens associated with BV, VVC, and STIs to inform individualized therapy and potentially reduce treatment failure or recurrence.

Although comparison between healthcare institutions was not the primary objective, clear and internally consistent differences were observed between the provincial CDC and the tertiary gynecology hospital. Women attending Hanoi OGH exhibited a higher symptom burden, particularly abnormal vaginal discharge (OR = 5.34), along with a markedly higher prevalence of elevated vaginal pH (OR = 10.0), and a substantially greater proportion of multi-pathogen infections (OR = 9.75) compared with women attending Bac Ninh CDC. These differences should be interpreted cautiously and primarily as reflections of healthcare-seeking behavior, referral pathways, and case mix, rather than as intrinsic epidemiological contrasts between institutions. Similar patterns have been described in prior studies, where tertiary centers disproportionately manage patients with persistent, recurrent, or treatment-refractory vaginitis [46,47]. From this perspective, healthcare setting functions as a proxy for disease complexity rather than a causal risk factor, reinforcing the generalizability of symptom- and pH-based associations across care levels.

Several limitations should be acknowledged. First, the cross-sectional design precludes causal inference and cannot determine whether multi-pathogen infection directly drives symptom severity or reflects cumulative effects of delayed care, prior treatment, or disease chronicity. Second, molecular detection identifies pathogen nucleic acids and does not distinguish between active infection and colonization. Third, microscopic staining and Nugent scoring were not included because these methods were not performed uniformly across the two study sites, potentially introducing inter-laboratory variability. Fourth, although healthcare setting was considered in secondary analyses, unmeasured differences in care-seeking behavior or prior antimicrobial exposure may have influenced observed patterns. Finally, while the sample size was defined by consecutive recruitment over a fixed study period and was adequate for the planned analyses, the facility-based design limits the generalizability of the findings to population-level prevalence estimates. Despite these limitations, the study provides real-world clinical and microbiological insights into symptom–pathogen associations in routine care facilities. Therefore, the findings of this study should be interpreted as hypothesis-generating rather than as population-representative epidemiological estimates.

## 5. Conclusions

In summary, this study demonstrates that infection complexity in symptomatic vaginitis is most strongly associated with abnormal vaginal discharge, itching, and elevated vaginal pH. Multi-pathogen infections represent a clinically meaningful phenotype, with *G. vaginalis* frequently acting as the central organism within polymicrobial combinations. Women attending the tertiary gynecology hospital show higher symptom burden, more frequent vaginal pH elevation, and a substantially greater prevalence of multi-pathogen infections than those presenting to the provincial CDC, likely reflecting differences in care-seeking behavior and referral pathways rather than intrinsic epidemiological variation. Taken together, these findings support a practical, severity-driven diagnostic approach integrating symptom clustering and vaginal pH to guide the rational use of multiplex testing, with particular relevance for tropical and resource-limited healthcare settings where optimized diagnostic allocation is essential. Future prospective studies incorporating longitudinal follow-up are needed to evaluate how symptom burden, vaginal pH, and molecularly defined infection complexity predict treatment response and recurrence over time.

## Figures and Tables

**Table 1:** Baseline demographic and clinical characteristics of women with vaginitis.

Characteristic	Total (*N* = 289)	Characteristic	Total (*N* = 289)
Age of participants		Genital hygiene practices *n* (%)	
Mean ± SD	31.01 ± 9.60	Vaginal douching	41 (14.19)
18–45 *n* (%)	264 (91.35)	Use of cleaning solution	225 (77.85)
46–60 *n* (%)	25 (8.65)	Clinical symptom *n* (%)	
Region *n* (%)		Abnormal vaginal odor	124 (42.91)
Urban	169 (58.48)	Abnormal vaginal discharge	257 (88.93)
Rural	120 (41.52)	Itching	188 (65.05)
Marital status *n* (%)		Dysuria/Frequent Urination	39 (13.49)
Single	118 (40.83)	Lower abdominal pain	37 (12.80)
Married	166 (57.44)	Symptom burden *n* (%)	
Divorced	4 (1.38)	Single symptom	37 (12.80)
Number of births *n* (%)		2 symptoms	104 (35.99)
No childbirth	128 (44.29)	≥3 symptoms	131 (45.33)
1 child	39 (13.49)	Vaginal pH *n* (%)	
≥2 children	122 (42.21)	3.8–4.5	61 (21.11)
History of HPV injection *n* (%)	51 (17.65)	>4.5	228 (78.89)

Values are shown as *n* (%), calculated from the total sample size (*N* = 289). SD: standard deviation; HPV: human papillomavirus.

**Table 2:** Vaginal pathogen distribution and infection complexity detected by multiplex real-time PCR.

Infection Category	Total (*N* = 289)	Infection Category	Total (*N* = 289)
Overall pathogen prevalence		*G. vaginalis*, *M. genitalium*	2 (0.69)
Bacterial Vaginosis *n* (%)		*G. vaginalis*, *C. albicans*	46 (15.92)
*Gardnerella vaginalis*	260 (89.97)	*G. vaginalis*, *C. trachomatis*	8 (2.77)
*Mycoplasma hominis*	28 (9.69)	*G. vaginalis*, HSV-1	1 (0.35)
Vulvovaginal Candidiasis *n* (%)		*T. vaginalis*, *M. hominis*	1 (0.35)
*Candida albicans*	63 (21.80)	3-pathogen infection	16 (5.54)
STIs *n* (%)		*G. vaginalis*, *C. albicans*, *M. hominis*	7 (2.42)
*Chlamydia trachomatis*	16 (5.54)	*G. vaginalis*, *C. albicans*, *C. trachomatis*	1 (0.35)
*Mycoplasma genitalium*	5 (1.73)	*G. vaginalis*, *C. albicans*, *T. vaginalis*	1 (0.35)
*Trichomonas vaginalis*	2 (0.69)	*G. vaginalis*, *C. albicans*, *M. genitalium*	1 (0.35)
*Neisseria gonorrhoeae*	3 (1.04)	*G. vaginalis*, *C. trachomatis*, *M. hominis*	2 (0.69)
Herpes simplex virus (HSV-1&2)	4 (1.38)	*G. vaginalis*, *C. trachomatis*, *N. gonorrhoeae*	1 (0.35)
Pathogen infection patterns		*G. vaginalis*, *M. hominis*, *M. genitalium*	1 (0.35)
Single-pathogen infection *n* (%)	180 (62.28)	*G. vaginalis*, *M. hominis*, HSV-1	1 (0.35)
*G. vaginalis*	173 (59.86)	*G. vaginalis*, *M. hominis*, *N. gonorrhoeae*	1 (0.35)
*M. hominis*	2 (0.69)	4-pathogen infection	3 (1.04)
*C. albicans*	4 (1.38)	*G. vaginalis*, *C. albicans*, *C. trachomatis*, *M. hominis*	1 (0.35)
HSV-1	1 (0.35)	*G. vaginalis*, *C. albicans*, *C. trachomatis*, *M. genitalium*	1 (0.35)
Multi-pathogen infection *n* (%)	88 (30.45)	*G. vaginalis*, *C. trachomatis*, *M. hominis*, *N. gonorrhoeae*	1 (0.35)
2-pathogen infection	68 (23.53)	5-pathogen infection	1 (0.35)
*G. vaginalis*, *M. hominis*	10 (3.46)	*G. vaginalis*, *C. albicans*, *C. trachomatis*, *M. hominis*, HSV-2	1 (0.35)

Values are shown as *n* (%), calculated from the total sample size (*N* = 289).

**Table 3:** Clinical factors associated with single and multi-pathogen vaginal infections.

Factor	Single Infection (*N* = 180)	Multi Infections (*N* = 88)	OR (95% CI)	*p* Value	Adjusted *p* Value
Clinical sign or symptom *n* (%)					
Abnormal vaginal odor	108 (60.00)	43 (48.86)	0.637 (0.3785–1.066)	0.0843 ^a^	0.1405
Abnormal vaginal discharge	151 (83.89)	85 (96.59)	5.442 (1.709–17.36)	0.0022 ^b^	0.011
Itching	108 (60.00)	67 (76.14)	2.127 (1.212–3.722)	0.0092 ^a^	0.023
Dysuria/Frequent Urination	22 (12.22)	15 (17.05)	1.476 (0.7215–3.001)	0.2824 ^a^	0.353
Lower abdominal pain	23 (12.78)	12 (13.64)	1.078 (0.5118–2.298)	0.8447 ^a^	0.8447
Symptom burden *n* (%)					
Single symptom	24 (13.33)	6 (6.82)	0.4756 (0.1959–1.203)	0.1121 ^a^	0.1716
2 symptoms	58 (32.22)	37 (42.05)	1.526 (0.7170–1.995)	0.1144 ^a^	0.1716
≥3 symptoms	82 (45.56)	44 (50.00)	1.195 (0.6396–1.829)	0.4936 ^a^	0.4936
Vaginal pH *n* (%)					
3.8–4.5	52 (28.89)	7 (7.95)	4.701 (2.110–11.26)	<0.0001 ^b^	
>4.5	128 (71.11)	81 (92.05)			

Notes: ^a^ Chi-square, ^b^ Fisher’s exact test.

**Table 4:** Clinical and vaginal infection factors associated with Bac Ninh CDC and Hanoi OGH.

Factor	Bac Ninh CDC (*N* = 128)	Hanoi OHG (*N* = 161)	OR (95% CI)	*p* Value	Adjusted *p* Value
Clinical sign or symptom *n* (%)					
Abnormal vaginal odor	91 (71.09)	64 (39.75)	0.2683 (0.1626–0.4395)	0.0001 ^a^	0.00025
Abnormal vaginal discharge	103 (80.47)	154 (95.65)	5.34 (2.301–13.53)	0.0001 ^a^	0.00025
Itching	80 (62.50)	108 (67.08)	1.223 (0.7453–2.002)	0.4172 ^a^	0.4172
Dysuria/Frequent Urination	13 (10.16)	26 (16.15)	1.704 (0.8644–3.415)	0.1386 ^a^	0.17325
Lower abdominal pain	12 (9.38)	25 (15.53)	1.777 (0.8711–3.628)	0.1199 ^a^	0.17325
Symptom burden *n* (%)					
Single symptom	12 (9.38)	25 (15.53)	1.777 (0.8711–3.628)	0.1199 ^a^	0.17985
2 symptoms	37 (28.91)	67 (41.61)	1.753 (1.067–2.871)	0.0254 ^a^	0.0762
≥3 symptoms	65 (50.78)	66 (40.99)	0.6734 (0.4253–1.09)	0.9669 ^a^	0.9669
Vaginal pH *n* (%)					
3.8–4.5	51 (39.84)	10 (6.21)	10.00 (4.905–20.07)	<0.0001 ^b^	
>4.5	77 (60.16)	151 (93.79)			
Infections					
Bacterial Vaginosis (BV) *n* (%)					
Gardnerella vaginalis	128 (100.00)	132 (81.99)	0.00 (0.000–0.1222)	<0.0001 ^b^	
Mycoplasma hominis	2 (1.56)	26 (16.15)	13.10 (3.495–56.71)	<0.0001 ^b^	
Vulvovaginal Candidiasis (VVC) *n* (%)					
Candida albicans	9 (7.03)	54 (33.54)	0.1499 (0.07102–0.3122)	<0.0001 ^a^	
Other STIs *n* (%)	0 (0.00)	30 (18.63)	0.00 (0.000–0.1169) ^c^	<0.0001 ^b^	
Chlamydia trachomatis	0 (0.00)	16 (9.94)	0.00 (0.000–0.2645) ^c^	<0.0001 ^b^	
Single or multi-infections *n* (%)					
Single infection	117 (91.41)	63 (39.13)	16.55 (8.289–32.92)	<0.0001 ^a^	
Multi-infections	11 (8.59)	77 (47.83)	9.75 (4.915–19.31)	<0.0001 ^a^	

Notes: ^a^ Chi-square, ^b^ Fisher’s exact test, ^c^ Baptista–Pike method.

## Data Availability

The datasets generated and analyzed during the current study contain individual-level clinical and microbiological data from human participants and therefore are not publicly available due to privacy and ethical restrictions approved by the Institutional Review Board. De-identified data may be made available for academic research purposes upon reasonable request to the corresponding author (vananhbiolab@gmail.com), subject to institutional approval and a data use agreement.

## Supplementary Materials

The following supporting information can be downloaded at: https://www.mdpi.com/article/10.3390/diagnostics16050783/s1, Table S1: Nucleotide sequences of primers and probes used for nine sexually transmitted infections (STIs) and the internal control in the three-panel real-time PCR assay; Table S2: Nucleotide sequences of the positive controls for the nine STIs and internal amplification control (IAC).
