# Supplementary material for: Clinical and Molecular Diagnostic Profiling of Vaginitis Using Multiplex Real-Time PCR: A Multicenter Study
Source: Diagnostics (Basel). 2026 Mar 5;16(5):783. doi: 10.3390/diagnostics16050783 (PMC12984344; doi:10.3390/diagnostics16050783)
Supplement: Supplementary file 1 [file diagnostics-16-00783-s001.zip › diagnostics-4146091-supplementary.docx]

**Supplementary Information**

**Table 1.** Nucleotide sequences of primers and probes used for nine sexually transmitted infections (STIs) and the internal control in the three-panel real-time PCR assay

| **Pathogen name** | **Primer/**  **Probe name** | **Sequence (5’-3’)** | **Length (bp)** | **T_m_ (℃)** | **Size (bp)** | **Gene name** |
| --- | --- | --- | --- | --- | --- | --- |
| ***Master mix 1*** |  |  |  |  |  |  |
| *G. vaginalis* | Fw-GV | GCCATTCTTGATGCCAATCG | 20 | 60.6 | 98 | *tuf* |
|  | Rv-GV | GGGTGTTGATTGGGAGCTTA | 20 | 60.7 |  |  |
|  | P-GV | /56FAM/TGTGTTCAC/ZEN/CATCTCCGGTCGTGGT/3IABkFQ/ | 25 | 69.8 |  |  |
| *N. gonorrhoeae* | Fw-NG | CAGCATTCAATTTGTTCCGAGTC | 23 | 61.3 | 91 | *porA pseudogene* |
|  | Rv-NG | CGGAACTGGTTTCATCTGATTAC | 23 | 60.2 |  |  |
|  | P-NG | /5HEX/ATACGCCTG/ZEN/CTACTTTCACGCTGG/3IABkFQ/ | 24 | 67.2 |  |  |
| *C. trachomatis* | Fw-CT | TTCAGTTGGGCCAGATCATG | 20 | 61.0 | 73 | *trpB* |
|  | Rv-CT | CTCTTCATCGGTGGCTAATGT | 21 | 60.8 |  |  |
|  | P-CT | /5Cy5/AAAGGCTCG/TAO/TCCTGACTCATGCAT//3IAbRQSp/ | 24 | 66.9 |  |  |
| ***Master mix 2*** |  |  |  |  |  |  |
| HSV-1 | Fw-HSV1/2 | GCAGTTTACGTACAACCACATACAGC | 26 | 64 | 117 | *UL27* |
|  | Rv-HSV1 | AGCTTGCGGGCCTCGTT | 17 | 63.4 |  |  |
|  | P-HSV1 | /56FAM/CGGCCCAAC/ZEN/ATATCGTTGACATGGC/3IABkFQ/ | 25 | 68.2 |  |  |
| *C. albicans* | Fw-CA | GTAGAAGGTCTGCTTCGTATGG | 22 | 60.8 | 100 | *RPR1* |
|  | Rv-CA | GTGACTTCAAGTTCGCATATTG | 22 | 58.8 |  |  |
|  | P-CA | /5HEX/CCGTGGATG/ZEN/GTTGGCTGTGAGTAA/3IABkFQ/ | 24 | 67.1 |  |  |
| *T. vaginalis* | Fw-TV | AACATTGACCACACGGACAA | 20 | 61.2 | 90 | *Repeated DNA target* |
|  | Rv-TV | CTTGGAACGTAAAGGCTTCTTC | 22 | 60.2 |  |  |
|  | P-TV | /5Cy5/TCATTTCGG/TAO/ATGGTC AGC AGCCA/3IAbRQSp/ | 23 | 67.1 |  |  |
| ***Master mix 3*** |  |  |  |  |  |  |
| *M. hominis* | Fw-MH | TTTGGTCAAGTCCTGCAACGA | 21 | 63.3 | 101 | *rrnB* |
|  | Rv-MH | CCCCACCTTCCTCCCAGTTA | 20 | 63.8 |  |  |
|  | P-MH | /56FAM/TACTAACAT/ZEN/TAAGTTGAGGACTCTA/3IABkFQ/ | 25 | 64.5 |  |  |
| HSV-2 | Fw-HSV1/2 | GCAGTTTACGTATAACCACATACAGC | 26 | 64.0 | 117 | *UL27* |
|  | Rv-HSV2 | AGCTTGCGGGCCTCGTT | 17 | 63.4 |  |  |
|  | P-HSV2 | /5HEX/CGCCCCAGC/ZEN/ATGTCGTTCACGT/3IABkFQ/ | 22 | 69.8 |  |  |
| *M. genitalium* | Fw-MG | TTATGCGCACCAGTTACTTG | 20 | 59.4 | 131 | *Hypothetical protein* |
|  | Rv-MG | AAGTTCAACTGCAGTAGTTGT | 21 | 59.0 |  |  |
|  | P-MG | /5Cy5/GGTGTGGAT/TAO/CGAGCGGC/3IAbRQSp/ | 17 | 63.4 |  |  |
| Internal Amplification Control (IAC) | Fw-IAC | TGAGCGCGGCTACAGCTT | 18 | 64.0 | 92 | *beta-actin* |
|  | Rv-IAC | TCCTTAATGTCACGCACGATTT | 22 | 61.6 |  |  |
|  | P-IAC | /5ROX/CCACCACGGCCGAGCGG//3IAbRQSp/ | 17 | 68.6 |  |  |

**Table 2.** Nucleotide sequences of the positive controls for the nine STIs and internal amplification control (IAC)

| **No.** | **PC name** | **Size (bp)** | **Sequence (5’-3’)** |
| --- | --- | --- | --- |
| 1 | *G. vaginalis* | 98 | GCCATTCTTGATGCCAATCGAAGATGTGTTCACCATCTCCGGTCGTGGTACCGTTGTCACCGGTCGTGTTGAGCGTGGTAAGCTCCCAATCAACACCC |
| 2 | *N. gonorrhoeae* | 91 | CAGCATTCAATTTGTTCCGAGTCAAAACAGCAAGTCCGCCTATACGCCTGCTACTTTCACGCTGGAAAGTAATCAGATGAAACCAGTTCCG |
| 3 | *C. trachomatis* | 73 | TTCAGTTGGGCCAGATCATGCCGAAATGCATGAGTCAGGACGAGCCTTTTATACATTAGCCACCGATGAAGAG |
| 4 | HSV-1 | 117 | GCAGTTTACGTACAACCACATACAGCGCCATGTCAACGATATGTTGGGCCGCGTTGCCATCGCGTGGTGCGAGCTGCAGAATCACGAGCTGACCCTGTGGAACGAGGCCCGCAAGCT |
| 5 | *C. albicans* | 100 | GTAGAAGGTCTGCTTCGTATGGGAATGGCGCCGTGGATGGTTGGCTGTGAGTAATTCTTTACTACAAGCTGTTTAGTGCAATATGCGAACTTGAAGTCAC |
| 6 | *T. vaginalis* | 90 | AACATTGACCACACGGACAAAAAGTGTCATTTCGGATGGTCAAGCAGCCAATCGCATTCGAGCACTTCGAAGAAGCCTTTACGTTCCAAG |
| 7 | *M. hominis* | 101 | TTTGGTCAAGTCCTGCAACGAGCGCAACCCCTATCTTTAGTTACTAACATTAAGTTGAGGACTCTAGAGATACTGCCTGGGTAACTGGGAGGAAGGTGGGG |
| 8 | HSV-2 | 117 | GCAGTTTACGTATAACCACATACAGCGCCACGTGAACGACATGCTGGGGCGCATCGCCGTCGCGTGGTGCGAGCTGCAGAACCACGAGCTGACTCTCTGGAACGAGGCCCGCAAGCT |
| 9 | *M. genitalium* | 131 | TTATGCGCACCAGTTACTTGAAAAAAATACCCATAATGAATAGTGATAGTGATCTAAAACTCCAAAAGGTGTGGATCGAGCGGCATGTTGATCAAGATGAACTTAGTTTAACAACTACTGCAGTTGAACTT |
| 10 | IAC | 92 | TGAGCGCGGCTACAGCTTCACCACCACGGCCGAGCGGGAAATCGTGCGTGACATTAAGGAAAGGGCGAATTCTGCAGATATCCATCACACTG |

***Reference***

1. Bui, H.T.V.; Bui, H.T.; Chu, S.V.; Nguyen, H.T.; Nguyen, A.T.V.; Truong, P.T.; Dang, T.T.H.; Nguyen, A.T.V. Simultaneous real-time PCR detection of nine prevalent sexually transmitted infections using a predesigned double-quenched TaqMan probe panel. *PLoS One* **2023**, *18*, e0282439.
